# In vitro assessment of the performance of a new multiband mucosectomy device for endoscopic resection of early upper gastrointestinal neoplasia

**DOI:** 10.1007/s00464-015-4222-x

**Published:** 2015-05-28

**Authors:** D. W. Schölvinck, K. Belghazi, R. E. Pouw, W. L. Curvers, B. L. A. M. Weusten, J. J. G. H. M. Bergman

**Affiliations:** Department of Gastroenterology and Hepatology, St. Antonius Hospital, Koekoekslaan 1, 3435 CM Nieuwegein, The Netherlands; Department of Gastroenterology and Hepatology, Academic Medical Center, Amsterdam, The Netherlands; Department of Gastroenterology and Hepatology, Catharina Hospital, Eindhoven, The Netherlands

**Keywords:** Endoscopic resection, Multiband mucosectomy, Esophageal neoplasia, Gastric neoplasia

## Abstract

**Background and study aims:**

Multiband mucosectomy (MBM) is widely used for the endoscopic resection of early neoplasia in the upper gastrointestinal tract. A new MBM-device may have advantages over the current MBM-device with improved visualization, easier passage of accessories, and higher suction power due to different trip wire and cap.

**Methods:**

Rubber bands were released one by one for both MBM-devices while endoscopic images were collected. First, free endoscopic view was assessed by computer-assisted measurements (quantitative) and by ranking the images by a panel of 11 endoscopists (qualitative). Second, using a visual analog scale, three ‘blinded’ endoscopists assessed introduction and advancement of three types of endoscopic devices through the working channel of a diagnostic endoscope with the MBM-devices assembled. Third, suction power was evaluated by a manometer attached to the cap of the assembled MBM-devices in four endoscopes. Negative pressures were measured after 5 and 10 s of suction and repeated five times. The passage and suction experiments were performed with dry trip wires and repeated after soaking with bloody, mucous fluids.

**Results:**

With all bands present, endoscopic views were 90 and 40 % in the new and current MBM-device, respectively. With the release of more bands, differences slowly disappeared. The panel scored a better endoscopic view in the new MBM-device (*p* = 0.03). Passage of all accessories was considered significantly easier in the new MBM-device. With the associated snare in the working channel, suction power was significantly better with the new MBM-device.

**Conclusion:**

Compared to the currently available MBM-device, the new MBM-device provides improved endoscopic visibility, smoother passage of accessories, and higher suction power.

Endoscopic resection (ER) is the cornerstone of endoscopic therapy of early esophageal neoplasia (i.e., high-grade dysplasia or early carcinoma). Histopathological assessment of the resection specimen provides the opportunity to determine whether further therapy is needed and, if so, to select patients suitable for further endoscopic treatment with additional ER or ablative therapy.

The ER-cap technique was the first widely used resection technique [1–3]. However, this procedure is technically demanding, particularly when multiple resections (i.e., piecemeal) are required. A more user-friendly alternative to the ER-cap method is the multiband mucosectomy (MBM) technique [4–7]. The currently available MBM-device utilizes a modified variceal band ligator with a control handle mounted at the proximal end of the working channel, connected by a trip wire to a transparent cap with six rubber bands that are placed on the tip of the endoscope. The target area is sucked into the cap without prior submucosal injection, followed by the release of a rubber band. The created pseudopolyp is subsequently resected using a hexagonal snare. This suck-band-snare procedure may be repeated six times per MBM-device.

In Barrett’s neoplasia, MBM achieves comparable success rates for effective piecemeal resection compared to the ER-cap technique, yet the procedure is quicker and cheaper [8–10]. Furthermore, complications rates (i.e., perforation or bleeding) are low, and most endoscopists consider the MBM technique easier to learn.

A disadvantage of the currently available MBM-device is somewhat restricted endoscopic visualization due to the presence of the black rubber bands on the distal attachment cap. Particularly, at the start of a MBM procedure, when all six bands are still present, these bands limit circumferential visualization within the endoscopic field. Reduced visualization may more easily lead to incomplete endoscopic resections and may hamper the management of complications such as bleeding.

Secondly, the fibrous structure of the trip wires used to release the bands of the currently available MBM-device causes it to swell when brought into contact with fluids such as water and blood. This further reduces the space within the working channel of the endoscope, which may interfere with the passage of accessories. It is possible that the swelling of the trip wires also results in a decreased suction pressure. An earlier in vitro study has demonstrated that a snare in the MBM-device (i.e., reducing the diameter of working channel) results in a decreased suction power [11]. Like the introduction of a snare, swelling of the trip wires by these liquids may (even further) reduce the diameter of the working channel and therefore limit the suction power, particularly in diagnostic endoscopes.

Recently, a new MBM-device has been developed with different trip wire and cap designs, which may overcome some of the disadvantages of the currently available MBM-device by improved visualization, easier passage of devices, and increased suction power. The aim of this study was to compare the visualization, ease of passage of endoscopic devices through the working channel, and suction power between the new and the currently available MBM-devices.

## Methods

### General materials

#### Multiband mucosectomy devices

**Fig. 1:**
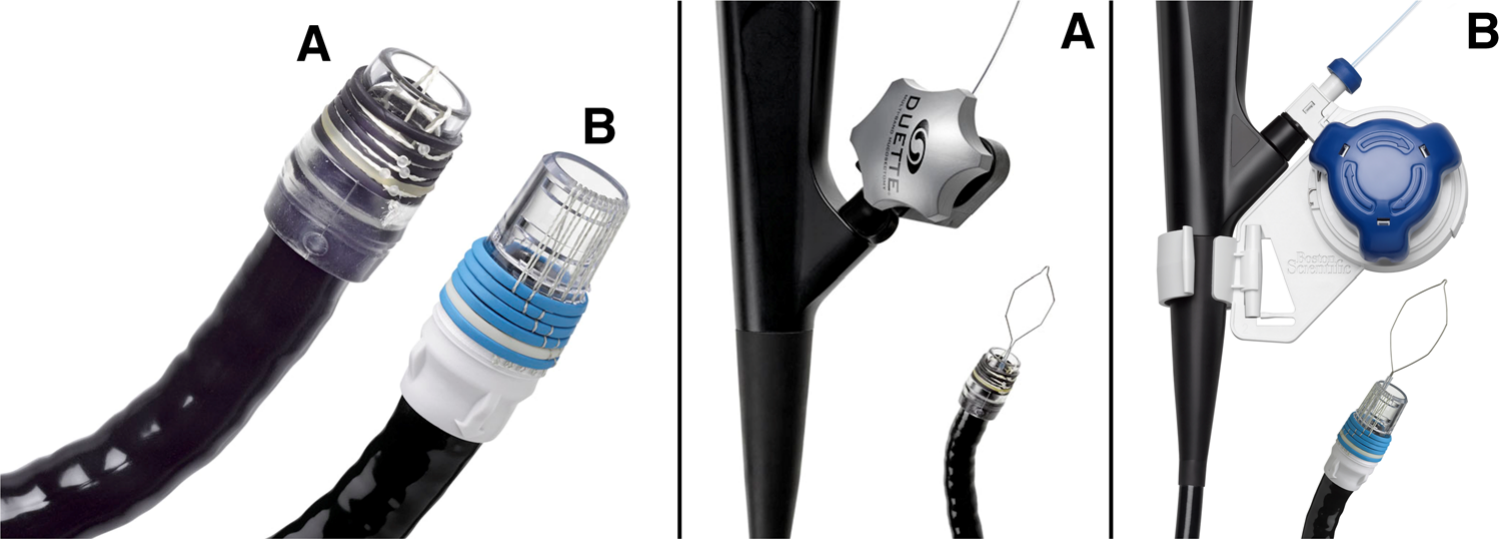
The new and current MBM-device. In the current MBM-device (**A**), the rubber bands are located at the distal side of the cap, which restricts the endoscopic view. By the location of the rubber bands at the proximal side of the cap, the new MBM-device (**B**) allows for a better endoscopic view

The new Captivator™ EMR device (Boston Scientific Corporation, Natick, MA, USA) consists of a plastic control handle, a metal trip wire, a transparent cap with six rubber bands mounted on the proximal side, and a 5-Fr (17 mm) stiff hexagonal snare (ø 1.8 mm). The currently available MBM-device (Duette™, DT-6-5F, Cook Medical, Limerick, Ireland) includes a control handle, a fibrous trip wire, a transparent cap with six rubber bands mounted on the distal side of the cap, and a 5-Fr hexagonal snare (ø 1.7 mm). In both devices, the control handle is assembled at the proximal end of the working channel through a closed biopsy hood. The trip wire is advanced through the working channel, followed by placement of the cap on the tip of the endoscope with the trip wire in correct position in the endoscopic field (Fig. 1). All of the following experiments were conducted with MBM-devices mounted as described above.

#### Endoscopes and other materials

In all experiments, diagnostic gastroscopes with a 2.8-mm-diameter working channel (GIF HQ190, Olympus, Hamburg, Germany) were used. In addition, three liters of fluids in suction bags from previous clinical upper gastrointestinal MBM procedures were collected and deep-frozen. Before running the experiments II and III, the collected fluids were thawed and heated up to body temperature (37 °C). During these experiments, the mucous and bloody fluids were suctioned at different time points to mimic endoscopic procedures in the in vivo situation.

### Experiment I: Visualization

The tip of the endoscope with the assembled MBM-devices was placed in a paper tube (ø 15 mm) with printed mucosa-colored background and a scale with 0.5-cm steps. This tube was used as a model ‘esophageal mucosa.’ Rubber bands were released one by one while high-quality images were obtained with the EXERA III processor system (Olympus Medical Systems, Europe, Hamburg, Germany) with settings at automatic light intensity. This resulted in six images per device.

**Fig. 2:**
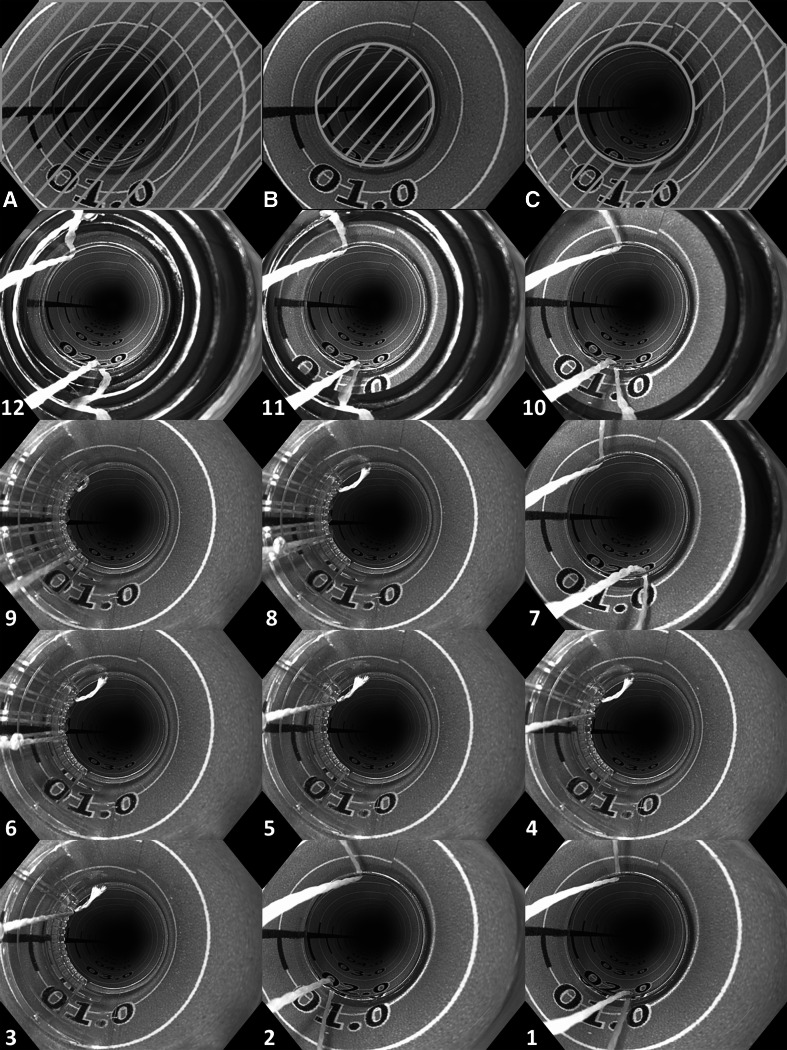
Endoscopic view with the new and current MBM-device. For the visualization experiment, rubber bands were released one by one while high-quality images were obtained in a paper tube with printed mucosa-colored background, resulting in six images per MBM-device. For quantitative analysis, the surface of visible ‘esophagus’ through the opening of the cap (**B**) as well as through the plastic wall of the cap (**C**) was delineated and calculated as a percentage of the total endoscopic field (**A**), using a software program (ImageJ 1.47k, National Institute of Health, USA). For qualitative assessment, the total of 12 endoscopic images were ranked by a panel of 11 endoscopists from ‘worst’ to ‘best’ visibility of the ‘esophageal mucosa.’ Images 12 (worst) to 1 (best) show the median ranked position as scored by the panel

For quantitative scoring, the total endoscopic image was considered 100 % of the endoscopic field (Fig. 2A). Next, the surface of visible ‘esophagus mucosa’ through the opening of the cap (Fig. 2B) as well as through the plastic wall of the cap (Fig. 2C) was delineated and calculated as a percentage of the total endoscopic field, using a software program (ImageJ 1.47k, National Institute of Health, USA).

For qualitative assessment, the total of 12 endoscopic images were presented in a randomized order to a panel of 11 endoscopists, familiar with ER procedures, but not involved in the recording of the endoscopic images of the model ‘esophagus mucosa.’ The panel was asked to rank the images from best to worst visibility of the ‘esophageal mucosa.’ Images were scored 1–12 for best to worst visualization.

Outcome parameters were the difference in visibility scored between the assembled new (Captivator™) and the currently available (Duette™) MBM-device after the one by one release of the rubber bands in a qualitative and quantitative manner.

### Experiment II: Passage of devices

**Fig. 3:**
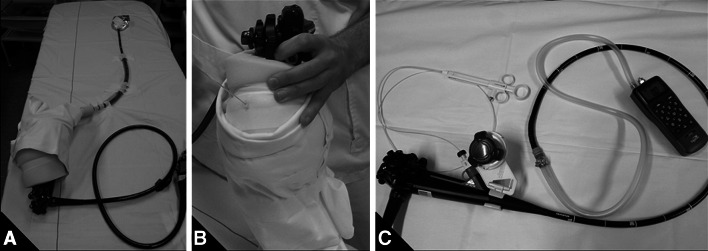
Setup for the ‘passage of devices’—and the ‘suction power’ experiment. During the ‘passage of devices’ experiment, the control handle and the cap of the assembled MBM-device were hidden to blind the panel of endoscopists (**A, B**). **C** The setup of the ‘suction power’ experiment: a manometer was attached by a plastic tube and metal ring to the distal end of the cap of the assembled MBM-device to measure the negative pressures of the vacuum system

For this experiment, two setups were prepared (Fig. 3): one gastroscope with the currently available MBM-device (setup 1) and one with the new MBM-device (setup 2). In order to mimic the position of the gastroscope in an in vivo situation, in this in vitro experiment the scope was locked in a bent position. To blind the endoscopists, the assembled MBM-devices were covered with foam rubber. Three endoscopists (B.W., J.B., and W.C.) introduced and advanced different endoscopic disposables [the associated snares of the devices, Resolution™ Clip Device (Boston Scientific Corporation, Natick, MA, USA), and the Interject Needle (Boston Scientific Corporation, Natick, MA, USA)] through the working channel until the tip of the endoscope was reached. The endoscopic device was first introduced in setup 1, followed by introduction in setup 2. The ease of introduction was scored on a visual analog scale (VAS score). This was repeated with the setups in reverse order. After soaking the dry releasing trip wires by suctioning the collected fluids multiple times (for 10 s at *t* = 0, *t* = 5, and *t* = 8 min), the whole experiment was repeated.

Outcome parameters were the number of times (proportion) the assembled new MBM-device or the current MBM-device was superior in ease of introduction of devices through the working channel with dry and soaked trip wires.

### Experiment III: Suction pressures

The suction pressure experiment was conducted in an endoscopy room (St. Antonius Hospital, Nieuwegein, The Netherlands) with a centrally regulated vacuum system for endoscopic suction (max 100 kPa) with the regulator at the vacuum connection fully open. Suction pressures were assessed by a manometer (2086p, Digitron, Torquay, UK) in kilopascal (kPa). With a silicone connection tube (ø 7 mm, 50 cm), the manometer was attached to the tip of the endoscope or to the MBM-device (tightened with a metal ring for optimal attachment).

Baseline suction pressures were measured at the tip of the gastroscope. Next, the negative pressure through the gastroscope was measured [1] with the assembled MBM-device with the trip wire in the working channel but without the snare and [2] with assembled MBM-device with trip wires and the snare in the working channel (i.e., with the trip wire plus snare in the working channel advanced until the tip of the endoscope was reached). Next, the collected liquids were suctioned multiple times (for 10 s at *t* = 0, *t* = 5, and *t* = 8 min). Measurements [1] and [2] were then repeated with a new assembled MBM-device, to prevent any leakage of the control handle due to earlier device introduction.

Negative pressures at the tip of the endoscope were measured five times, after 5 and 10 s of continuous maximum suction. To prevent influence of outliers, all measurements were taken with four different diagnostic gastroscopes.

The primary outcome parameter was the mean pressure difference between the new and the currently available MBM-device assembled on a diagnostic scope with dry trip wires after 5 and 10 s of suction. Secondary outcome parameters were the influence of soaking of the wires on the mean pressure difference between the new and the current MBM-device and the influence of a snare in the working channel for the new and the current MBM-device on mean pressure difference in case of dry trip wires and soaked trip wires.

### Statistical analysis

For this in vitro bench test study, no power analysis or sample size calculation was performed since data on point estimates of the variables of interest were not available from the literature. Statistical analysis was performed with the Statistical Software Package version 20.0.0.1 for windows (SPSS, Chicago, Illinois, USA). For descriptive statistics, mean with standard deviation was used for variables with a normal distribution and the median with interquartile range (IQR) was used for variables with a skewed distribution. For experiment I (visualization), the ranked images of the new and currently available MBM-device were compared using a Wilcoxon signed rank test. For experiment II (passage of devices), the middle of the VAS scale was considered ‘0 %,’ extending to either ‘−100 %’ for the current MBM-device or ‘+100 %’ for the new MBM-device. One-sample *t* test allowed for comparison of the preference percentage to the reference point ‘0.’ In experiment III (suction pressures), comparison of suction pressures was performed with linear mixed model analysis to correct for clustering of measurements within endoscopes.

## Results

### Experiment I: Visualization

Endoscopic visualization through the opening of the cap was 15 and 20 % of the total endoscopic view for the new and the current MBM-device, respectively. Table 1 shows the visualization through the plastic of the caps for both MBM-devices: With all rubber bands present, limited visualization is allowed through the plastic of the currently available MBM-device (20 %) compared with the new MBM-device (74 %), but visualization improves with each release of a rubber band. When combining the visualization through the opening and the plastic of the cap, the new MBM-device allows visualization of 90 % of the endoscopic view directly after mounting the device. With the current MBM-device, visualization of >90 % of the total endoscopic view is not reached until releasing 4 of the 6 rubber bands.

**Table 1:** Quantitative analyses of the endoscopic view with the new and the current MBM-device

Number of bands	Circumferential visualization through the opening and the plastic wall of the cap (of total endoscopic view), %		Circumferential visualization through the plastic wall of the cap (of total endoscopic view), %	
	New MBM-device	Current MBM-device	New MBM-device	Current MBM-device
All bands	90	40	74	20
1 Released	91	53	76	33
2 Released	93	69	77	49
3 Released	94	80	78	60
4 Released	95	92	79	72
5 Released	96	95	80	75
All bands released	97	100	82	80

For quantitative analyses, the endoscopic view through the opening of the cap and through the plastic wall of the cap was measured with a software program (ImageJ 1.47k, National Institute of Health, USA) and calculated as a percentage of the total endoscopic image (see also Fig. 2A–C). Endoscopic view is limited by the wires, rubber bands (current MBM-device), and the jagged rim (new MBM-device). After release of the fourth band, the endoscopic view becomes comparable between both MBM-devices

The median order in which the images were ranked by the panel for qualitative analysis is displayed in Fig. 2A–L, with a score of ‘1’ being the best and ‘12’ being the worst. The overall median score for the visualization with the new MBM-device was 5.5 (5.5–6.5), whereas this was 8.5 (6.5–9.5) for the current MBM-device (*p* = 0.03). As shown in Table 2, better visualization with the new MBM-device compared to the current MBM-device is mainly present until the fourth band is released, after which the trend is reversed.

**Table 2:** Qualitative analyses of the endoscopic view with the new and the current MBM-device

Number of bands	Median (IQR) ranking scores		*p* value
	New MBM-device	Current MBM-device	
All bands	9 (8–10)	12 (12–12)	<0.001
1 Released	8 (7–9)	11 (11–11)	<0.001
2 Released	6 (6–7)	10 (8–10)	<0.001
3 Released	5 (5–6)	7 (5–9)	0.025
4 Released	4 (4–4)	2 (2–2)	<0.001
5 Released	3 (3–3)	1 (1–1)	<0.001
Overall	5.5 (5.5–6.5)	8.5 (6.5–9.5)	0.03

The total of 12 different images, with rubber bands released one by one (six images with each MBM-device), were ranked according to the ‘best’ (1) to ‘worst’ (12) endoscopic view. The new MBM-device was considered to have a better endoscopic view than the current MBM-device until the fourth band was released

*IQR* interquartile range

### Experiment II: Passage of devices

**Fig. 4:**
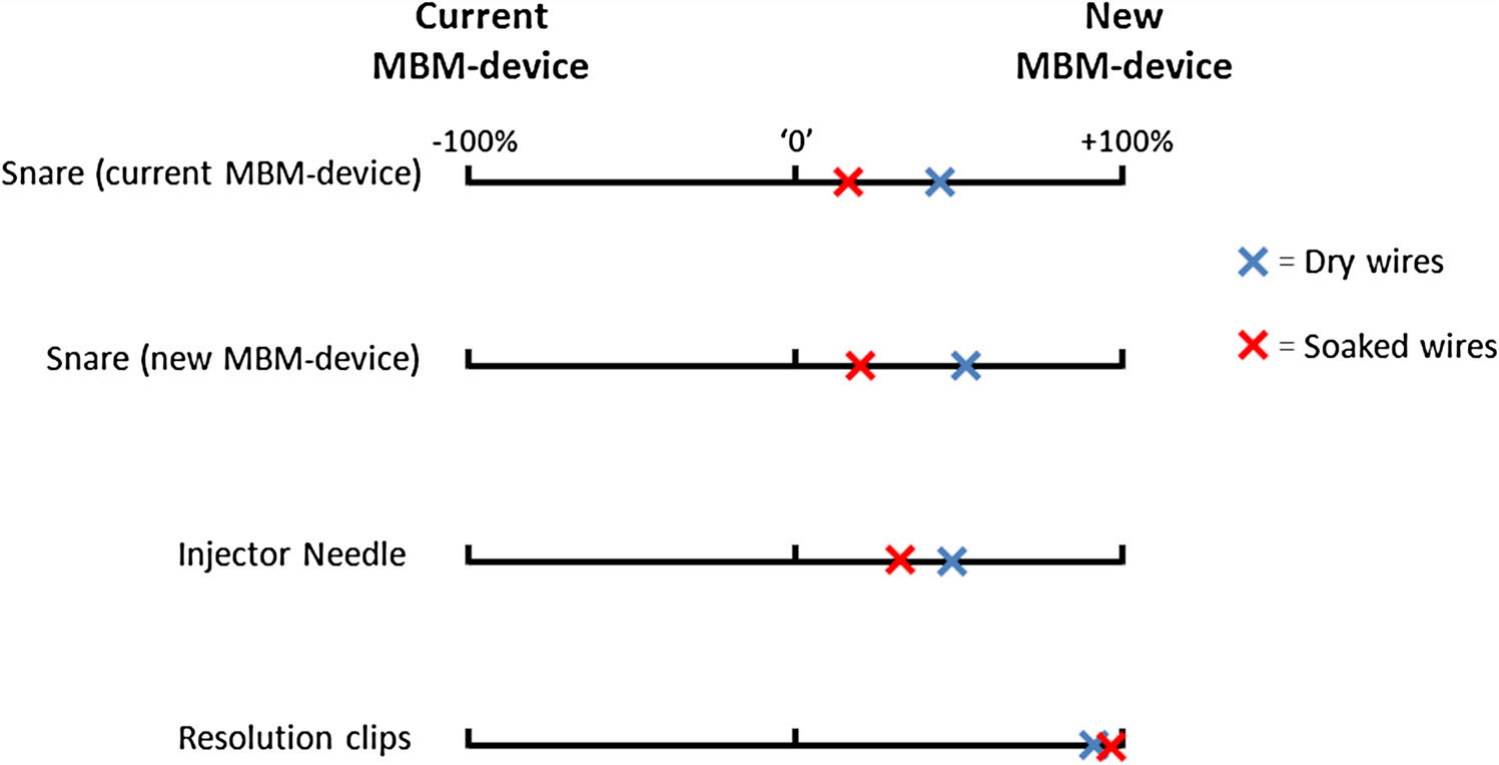
Scores of the ‘passage of devices’ experiment. These scales show the median preference of the panel of blinded endoscopists for the introduction and advancement of different endoscopic devices through the working channel of a diagnostic endoscope with the assembled new and the current MBM-device. ‘0’ represents the reference point where the new MBM-device and current MBM-device would be considered equal. This experiment was performed with dry trigger cords and repeated after soaking the trigger cords with mucous and bloody fluids

Figure 4 shows the average scores on the VAS scale of the three endoscopists per endoscopic device that was introduced and advanced through the working channel of a diagnostic endoscope. Table 3 shows the average scores in numbers as displayed in Fig. 4. All devices did pass significantly more smoothly with the assembled new MBM-device compared to the currently available MBM-device. Advancement of devices along the dry trip wires appeared more easy than with soaked trip wires, however, only significant during advancement of the snare associated with the new MBM-device (*p* = 0.03).

**Table 3:** Ease of passage of endoscopic devices through the working channel of a diagnostic gastroscope mounted with either the new MBM-device or the current MBM-device

Situation	Device	Percentage from point where the new and current MBM-device are considered equal Mean (±SD)	*p* value
Dry wires	Snare (current MBM-device)	+43 % (±30)	0.017
	Snare (new MBM-device)	+50 % (±24)	0.003
	Injector needle	+49 % (±11)	<0.001
	Resolution clips	+89 % (±13)	0.001
Soaked wires	Snare (current MBM-device)	+15 % (±14)	0.042
	Snare (new MBM-device)	+19 % (±14)	0.019
	Injector needle	+30 % (±17)	0.006
	Resolution clips	+96 % (±9)	<0.001

Positive mean percentages point toward easier introduction with the new MBM-device, negative mean percentages point toward easier introduction with the current MBM-device. *p* values represent the difference of the average score to the reference point ‘0,’ where the new and current MBM-device would be considered equal (Fig. 4). For all endoscopic devices, the passage was considered much easier in the new MBM-device when assembled on a diagnostic endoscope (GIF HQ190, Olympus, Hamburg, Germany), with or without the trip wires soaked in mucous and bloody fluids

*SD* standard deviation

### Experiment III: Suction experiment

The mean (±SD) baseline negative pressure measurements at the tip of the endoscope without assembled MBM-devices of the four setups were 44 (±1.6) and 52 (±0.6) kPa after 5 and 10 s, respectively. With the new and the current MBM-device assembled at the tip of the endoscope, negative pressures did not significantly differ at 5 s (mean 42.3 and 41.7 kPa, respectively; *p* = 0.42) and 10 s (mean 50.7 and 49.8 kPa, respectively; *p* = 0.15). After advancement of the associated snare, a loss of negative pressure was observed. Significantly, less suction power was available with the current MBM-device compared to the new MBM-device at 5 s (mean 26.4 and 28.8 kPa, respectively; *p* = 0.04) and 10 s (mean 29.8 and 32.8 kPa, respectively; *p* = 0.02). After soaking the wires in mucous fluids, the negative pressures in both setups, MBM-no-snare and MBM-with-snare, decreased slightly. In the MBM-with-snare setup, the difference between the new and the current MBM-device was still present, yet no longer statistically significant (Table 4).

**Table 4:** Suction power with the assembled new and the current MBM-device

	5 s			10 s		
	New MBM-device	Current MBM-device	*p* value	New MBM-device	Current MBM-device	*p* value
	Mean (95 % CI) Pressure (kPa)	Mean (95 % CI) Pressure (kPa)		Mean (95 % CI) Pressure (kPa)	Mean (95 % CI) Pressure (kPa)	
*Dry wires*						
MBM-device, no snare	42.3 (41.3–43.3)	41.7 (40.7–42.7)	0.42	50.7 (49.8–51.6)	49.8 (48.9–50.7)	0.15
MBM-device and snare	28.8 (27.2–30.5)	26.4 (24.7–28.0)	0.04	32.8 (31.1–34.4)	29.8 (28.1–31.5)	0.02
*Soaked wires*						
MBM-device, no snare	40.7 (39.1–42.3)	40.2 (38.6–41.8)	0.65	49.6 (48.6–50.6)	48.0 (47.0–49.0)	0.03
MBM-device and snare	27.1 (25.2–29.1)	24.5 (22.6–26.5)	0.06	32.1 (30.2–34.1)	29.5 (27.6–31.5)	0.06

Linear mixed model analysis was performed to correct for clustering of measurements within endoscopes. Estimated marginal means with 95 % confidence intervals are shown. Compared to baseline [mean 44 (±SD 1.6) kPa and 52 (±0.6) kPa after 5 and 10 s, respectively], negative pressure did not decrease much after assembling of both MBM-devices. Introduction of the associated snare resulted in lower pressures. Negative pressures dropped a little more in the current MBM-device than in the new MBM-device, resulting in a significant difference in suction power. Soaking of the trigger cords with mucous and bloody fluids did not further increase the differences

*CI* confidence interval, *kPa* kilopascal, *MBM* multiband mucosectomy

## Discussion

Multiband mucosectomy is a widely used technique for the endoscopic resection of neoplasia in the upper gastrointestinal tract. MBM is cheaper and quicker than ER-cap technique, and complication rates are low [8–10]. In this in vitro study, we found that the new MBM-device allows for improved endoscopic visibility, easier passage of endoscopic devices, and marginally improved suction power compared to the currently used MBM-device. These improved features may facilitate easier and safer endoscopic resection with the ‘suck-and-ligate method.’

The rubber bands at the distal end of the cap of the currently available MBM-device restrict the endoscopic view. The rubber bands of the new MBM-device are located at the proximal side of the cap and outside the endoscopic view. The two visualization experiments show that this is associated with a significantly better endoscopic view until the release of the fourth band, which is of relevance since the majority of ER procedures requires no more than three or four bands [9]. The cap of the new MBM-device still has a small area where the endoscopic view is slightly impaired due to the semitransparent anchoring site of the releasing wire. This site is, however, relatively small, and its position within the endoscopic field can easily be changed by turning the scope (anti-)clockwise.

A good endoscopic view is important for several reasons. First, ER is considered endoscopically radical when all coagulation markers, placed to delineate the neoplastic lesion with a margin, have been removed by the resection. A better endoscopic view allows for easier targeting of these delineation markers. Second, safety of the MBM procedure may be increased since improved endoscopic view may achieve easier detection and approach of bleeding sites that may occur during endoscopic resection [9].

In addition, easy access with hemostatic devices improves the practicality of managing bleedings during endoscopic resection procedures. The current study shows that the introduction and advancement of endoscopic disposables is easier in the new MBM-device compared to the currently available MBM-device. This probably reflects the relatively thick and rough-textured fibrous trip wires of the current MBM-device, whereas the trip wire of the new MBM-device uses a relatively thin, smooth-textured metal wire to release the bands.

By soaking the working channel of the endoscope with mucous and bloody fluids, we expected swelling of fibrous textured trip wires of the current MBM-device, leading to further narrowing of the lumen of the working channel. Under these circumstances, however, the difference in scores for the advancement of the disposables between both MBM-devices became less pronounced. Apparently, the soaking smoothens the texture of the fibrous trip wires in the current MBM-device, thereby allowing easier passage. Nevertheless, under all tested circumstances the introduction and advancement of accessories was found to be easier in the new MBM-device.

During most clinical MBM procedures, the snare, which is used to resect the pseudopolyp after the band ligation, is kept inside the working channel of the endoscope throughout the procedure. Our suction power experiment shows that under these circumstances the suction power is significantly higher with the new MBM-device. With the current MBM-device, the working channel is substantially more narrowed by the combination of the snare with fibrous trip wires compared to the snare and the metal trip wires of the new MBM-device. After soaking of the working channel with mucous and bloody fluids, there was still a pressure difference in favor of the new MBM-device, yet this did not reach statistical significance.

Although decreased suction power may influence the size of a resection specimen as well as the ability to clear the endoscopic field of fluids and blood, the maximum difference of 3 kPa, observed between the two devices, is relatively small. An earlier study found differences in negative pressure up to 8 kPa when setups were tested in multiple endoscopy rooms [11]. Apparently, this difference in vacuum power occurs in daily practice and remains unnoticed or is not considered disturbing. Moreover, in daily practice suction of mucosa into the cap is achieved in less than 5 s (used in this study). This suggests that relatively low negative pressures are required for successful band ligation.

Our study has some limitations that need to be addressed. First, MBM-devices were evaluated in vitro, and results may be different in an in vivo situation. For example, in vivo assessment of visualization may be influenced by the texture of the mucosa and scattering of light, which we cannot simulate in a paper tube. To mimic the in vivo situation as much as possible, in the ‘passage of devices’ experiment, endoscopes were orientated according to the human anatomy during gastroscopic procedures. In addition, in ‘passage of devices’ and the ‘suction power’ experiment, suctioning of mucous and bloody fluids at body temperature was performed.

Second, some parameters in this study were subjective measures. Endoscopists were blinded to the type of device in the ‘passage of devices’ experiment, but in the endoscopic visualization experiments blinding was impossible as the devices were visible in the endoscopic view. To prevent the order of presentation of images to influence the visibility scoring, images were presented in a randomized order. Moreover, the results from the objective and subjective visualization experiment appear to be consistent.

Third, all experiments in this study were conducted with diagnostic endoscopes. These gastroscopes have a small caliber working channel (2.8-mm ID) but allow for optimal endoscopic imaging before, during and after the MBM procedure. In our unit, diagnostic gastroscopes are preferred for all MBM procedures. Some endoscopists may, however, prefer to use therapeutic endoscopes for performing MBM. Under these circumstances, the larger working channel of the therapeutic gastroscope may eliminate the observed differences in passage of devices, yet the difference in visualization in favor of the new MBM-device will likely remain unchanged.

In conclusion, compared to the currently available MBM-device, the new MBM-device is associated with improved endoscopic visibility, smoother passage of endoscopic devices, and marginally improved suction power. These features may improve the practicality of MBM. Clinical studies are, however, warranted to assess the safety, feasibility, and efficacy of the device in an in vivo setting.

## Abbreviations

ER: Endoscopic resection
MBM: Multiband mucosectomy
VAS: Visual analog scale

## References

[1] Inoue H, Takeshita K, Hori H, Muraoka Y, Yoneshima H, Endo M (1993). Endoscopic mucosal resection with a cap-fitted panendoscope for esophagus, stomach, and colon mucosal lesions. Gastrointest Endosc.

[2] Ell C, May A, Gossner L, Pech O, Gunter E, Mayer G, Henrich R, Vieth M, Muller H, Seitz G, Stolte M (2000). Endoscopic mucosal resection of early cancer and high-grade dysplasia in Barrett’s esophagus. Gastroenterology.

[3] Peters FP, Brakenhoff KP, Curvers WL, Rosmolen WD, ten Kate FJ, Krishnadath KK, Fockens P, Bergman JJ (2007). Endoscopic cap resection for treatment of early Barrett’s neoplasia is safe: a prospective analysis of acute and early complications in 216 procedures. Dis Esophagus.

[4] Soehendra N, Seewald S, Groth S, Omar S, Seitz U, Zhong Y, de Weerth A, Thonke F, Schroeder S (2006). Use of modified multiband ligator facilitates circumferential EMR in Barrett’s esophagus (with video). Gastrointest Endosc.

[5] Seewald S, Ang TL, Omar S, Groth S, Dy F, Zhong Y, Seitz U, Thonke F, Yekebas E, Izbicki J, Soehendra N (2006). Endoscopic mucosal resection of early esophageal squamous cell cancer using the Duette mucosectomy kit. Endoscopy.

[6] Peters FP, Kara MA, Curvers WL, Rosmolen WD, Fockens P, Krishnadath KK, Ten Kate FJ, Bergman JJ (2007). Multiband mucosectomy for endoscopic resection of Barrett’s esophagus: feasibility study with matched historical controls. Eur J Gastroenterol Hepatol.

[7] Pouw RE, Bergman JJ (2008). Endoscopic resection of early oesophageal and gastric neoplasia. Best Pract Res Clin Gastroenterol.

[8] Pouw RE, van Vilsteren FG, Peters FP, Alvarez Herrero L, Ten Kate FJ, Visser M, Schenk BE, Schoon EJ, Peters FT, Houben M, Bisschops R, Weusten BL, Bergman JJ (2011). Randomized trial on endoscopic resection-cap versus multiband mucosectomy for piecemeal endoscopic resection of early Barrett’s neoplasia. Gastrointest Endosc.

[9] Alvarez Herrero L, Pouw RE, van Vilsteren FG, ten Kate FJ, Visser M, Seldenrijk CA, van Berge Henegouwen MI, Weusten BL, Bergman JJ (2011). Safety and efficacy of multiband mucosectomy in 1060 resections in Barrett’s esophagus. Endoscopy.

[10] May A, Gossner L, Behrens A, Kohnen R, Vieth M, Stolte M, Ell C (2003). A prospective randomized trial of two different endoscopic resection techniques for early stage cancer of the esophagus. Gastrointest Endosc.

[11] Alvarez Herrero L, Mijnals C, Grimbergen CA, Weusten BL, Bergman JJ (2012) Influence of multi-band mucosectomy assembly and snare on endoscopic suction pressure of gastroscopes. St. Antonius Hospital, Nieuwegein (unpublished manuscript)

